# ECMO-weaning facilitated by neurally adjusted ventilatory assist (NAVA): a case for principal clarification

**DOI:** 10.1007/s10047-024-01484-6

**Published:** 2024-12-13

**Authors:** F. Heinold, O. Moerer, L. O. Harnisch

**Affiliations:** Department of Anaesthesiology, University Medical Center Göttingen, Robert-Koch-Str. 40, 37075 Göttingen, Germany

**Keywords:** Thoracic emergencies, Extracorporeal life support (ECLS), Veno-venous extracorporeal membrane oxygenation (vv-ECMO), Neurally Adjusted Ventilatory Assist (NAVA)

## Abstract

The use of veno-venous extracorporeal membrane oxygenation (VV-ECMO) has become increasingly prevalent, particularly in respiratory disease pandemics such as H1N1-influenza and SARS-CoV-2. This surge has emphasized the importance of clear therapy recommendations, improved accessibility to ECMO technology, established ECMO teams, and structured networks to ensure access to specialized care throughout the course of the disease for patients with severe ARDS. Although the initiation criteria for VV-ECMO are well defined, treatment strategies while on ECMO regarding e.g., ventilator management or ECMO weaning strategies remain variable and with lack of consensus. NAVA (Neurally Adjusted Ventilatory Assist), as an assisted mechanical ventilation modality, offers real-time electromyographic feedback, which has been shown to enhance prolonged weaning processes from mechanical ventilation. We present a case of penetrating thoracic trauma complicated by ARDS, successfully managed with VV-ECMO. NAVA was employed to monitor and facilitate ECMO. This approach integrates ECMO weaning with ventilation settings, considering both gas exchange lung function, such as carbon dioxide removal, and respiratory mechanics in the form of neuromuscular coupling. This is a new approach to VV-ECMO weaning. More research is planned to validate the efficacy of this method in conjunction with additional parameters, such as diaphragm activity evaluated sonographically in a randomized design. This case underscores the potential of NAVA in VV-ECMO weaning, offering a promising avenue for optimizing patient care and outcomes.

**Supplementary Information:**

The online version contains supplementary material available at 10.1007/s10047-024-01484-6.

## Background

The use of venovenous extracorporeal membrane oxygenation has increased in recent years and was undoubtedly boosted by pandemics of respiratory diseases such as H1N1-influenza and SARS-CoV-2 [20, 21, 26]. Despite this increased use, which is partially due to the increased availability of this method even in small hospitals, the recommendations are clear that VV-ECMO is still a rescue measure for the most severe cases that cannot be handled otherwise. The presence of established ECMO teams and the development of structured networks have contributed to ensuring that patients with severe ARDS have access to specialized care in ARDS centers, preventing avoidable mortality due to supply or expertise shortages.

The indications to initiate VV-ECMO independently of the cause of respiratory failure are fairly clear, but contraindications remain a matter of debate [11]. This is also true for ventilator and extracorporeal treatment strategies while on ECMO and especially with respect to the concept of weaning patients from the ECMO circuit and the ventilator [1, 4, 11]. Regarding the VV-ECMO weaning process, there are no generally accepted recommendations or much scientific evidence on how to make this process more meaningful.

Mechanical ventilation weaning is challenging for up to 30% of critically ill patients [12] with prolonged weaning is associated with increased morbidity, mortality, longer hospital stay, and risk of discharge to a long-term care facility [19]. Weaning from mechanical ventilation is known to be influenced by the mode of ventilation used; (partially) automated and/or adaptive ventilation modes may be advantageous in this regard [17]. NAVA (Neurally Adjusted Ventilatory Assist) was established in 2007 as a mode of assisted mechanical ventilation that uses the output of the patient’s respiratory center, which is tapped as a diaphragm myogram, to regulate the ventilator. The electrical signal that triggers the diaphragm is acquired via a specialized gastric feeding tube (Edi catheter). The Edi catheter is inserted into the esophagus close to the crural diaphragm to capture the neuronal excitations of the diaphragm [2, 17, 18, 24]. In real time, the recorded signal represents the temporal and spatial sum of motor unit recruitment and firing frequency [6, 15, 18]. The Edi peak refers to the highest electrical signal from the diaphragm during a breath cycle, indicating the level of respiratory effort. A higher Edi peak suggests increased breathing effort, signaling that the ventilator may need to provide more assistance. This Edi signal is multiplied by a user-controlled gain factor, the NAVA level (comparable to the level of pressure support in pressure support ventilation; unit: mbar/µV) which determines the level of applied airway pressure [3, 24]. Thus, the pressure support is directly proportional to the amplitude and duration of the Edi signal. When the NAVA level is increased, an instantaneous increase in pressure support is induced, leading to presumably more effective relief of the respiratory muscles [24]. A systematic increase in the level of NAVA has been shown to relieve inspiratory muscles in 74% of cases [3]. As a result, NAVA is frequently used to assess diaphragmatic activity and facilitate prolonged weaning, but has only been mentioned sporadically as an option in the context of ECMO therapy [14, 16].

We present a case of penetrating thoracic trauma complicated by ARDS, in which the patient not only underwent successful treatment with VV-ECMO, but also had the removal of the ECMO circuit monitored using NAVA. Although several studies have documented the use of extracorporeal membrane oxygenation in trauma patients, achieving survival rates ranging from approximately 44 to 71%, it is notable that patients with blunt thoracic trauma are significantly more likely to require ECMO therapy compared to those with penetrating thoracic trauma [8, 9].

## Case presentation

**Fig. 1:**
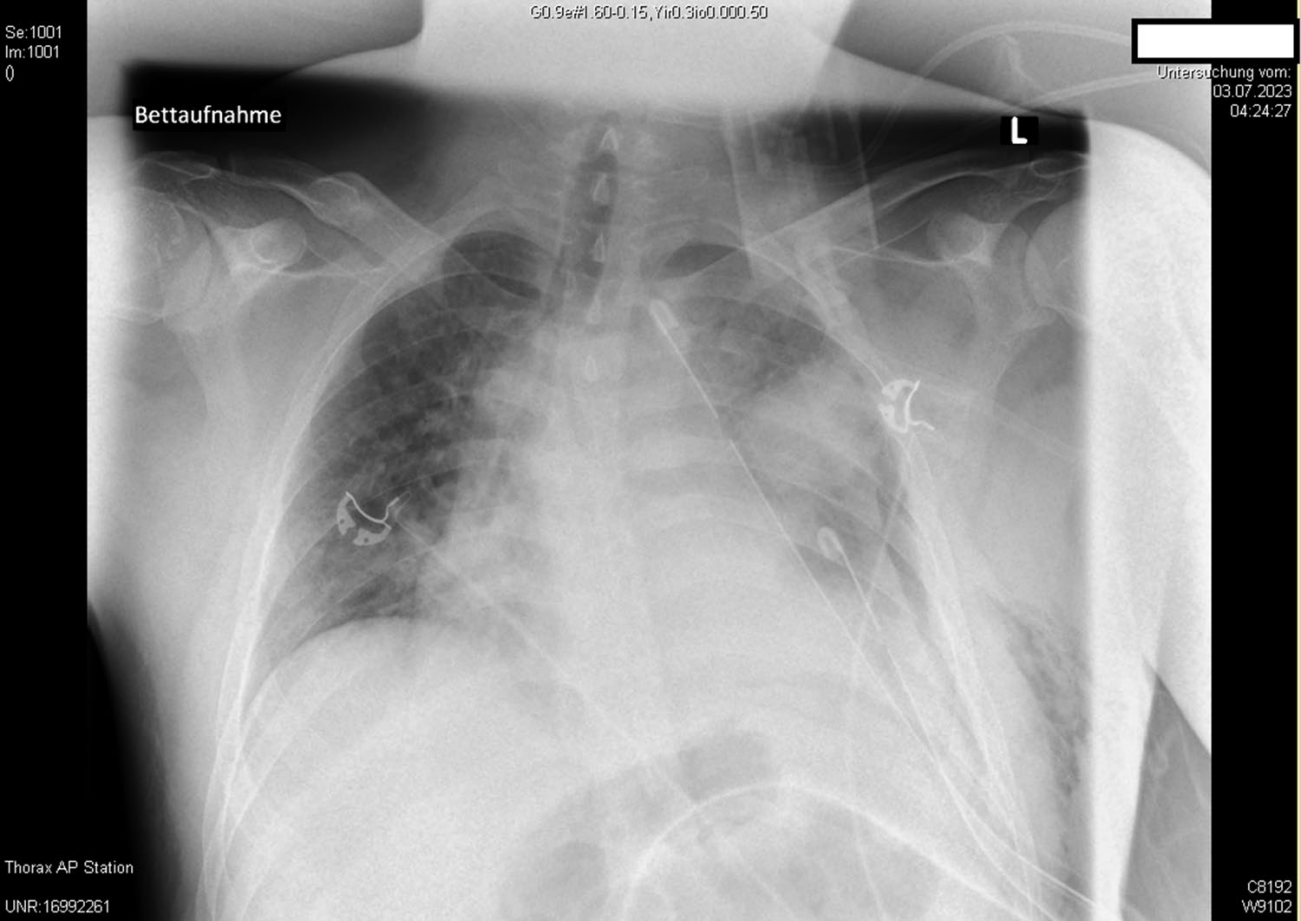
Chest radiography upon ICU admission, with two indwelling left sided chest drains in place, in the presence of persistent left sided pneumo- and hemothorax

A 27-year-old patient suffered a penetrating thoracic injury from a knife attack and was transferred to our trauma center for specialized surgical and ICU treatment. At the time of admission, the patient was fully conscious and did not have motor deficits, respiration was compensated with two chest drains in place, but still a verifiable hemato-pneumothorax on chest radiograph (Fig. 1 Chest radiography upon ICU admission, with two indwelling left-sided chest drains in place, in the presence of persistent left sided pneumo- and hemothorax.); otherwise, the patient was in good and stable condition.

Three days after admission, the patient developed severe pneumonia and had to be intubated and ventilated. The remaining hematoma, not fully relieved by thoracic drains, was treated by video-assisted thoracoscopic surgery (VATS) the following day. Despite these interventions, as well as extended antibiotic coverage and adjunctive measures, the patient’s respiratory condition further deteriorated to severe acute respiratory distress syndrome (ARDS) and conservative treatment options were exhausted. Therefore, VV-ECMO (Maquet-Cardiohelp, Version 1, HLS Set Advanced 7.0; 23 Fr. V. jugularis interna (Inflow), 25 Fr. V. femoralis sinistra (Outflow)) was initiated as a rescue measure according to criteria established in the EOLIA-trial (PaO2/FIO2 < 50 mmHg at FIO2 ≥ 80% for > 3 h despite the optimization of ventilation and the duration of mechanical ventilation ≤ 6 days) [5]. The initial settings included a pump flow of 5 L/min and a sweep-gas flow of 10 L/min. Tracheostomy had already been performed.

**Fig. 2:**
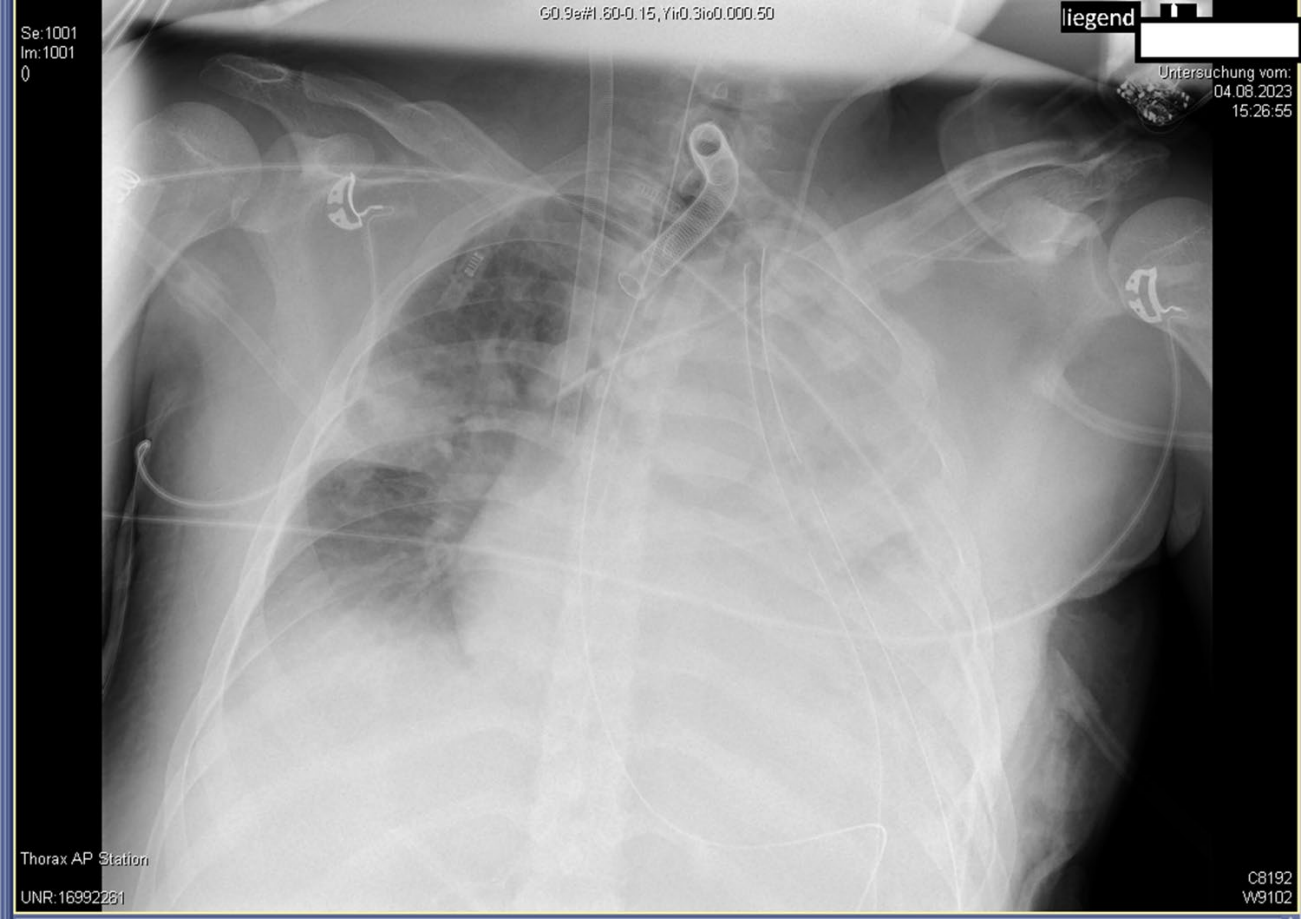
Chest radiography following ECMO and Tracheostomy tube placement in ARDS

**Fig. 3:**
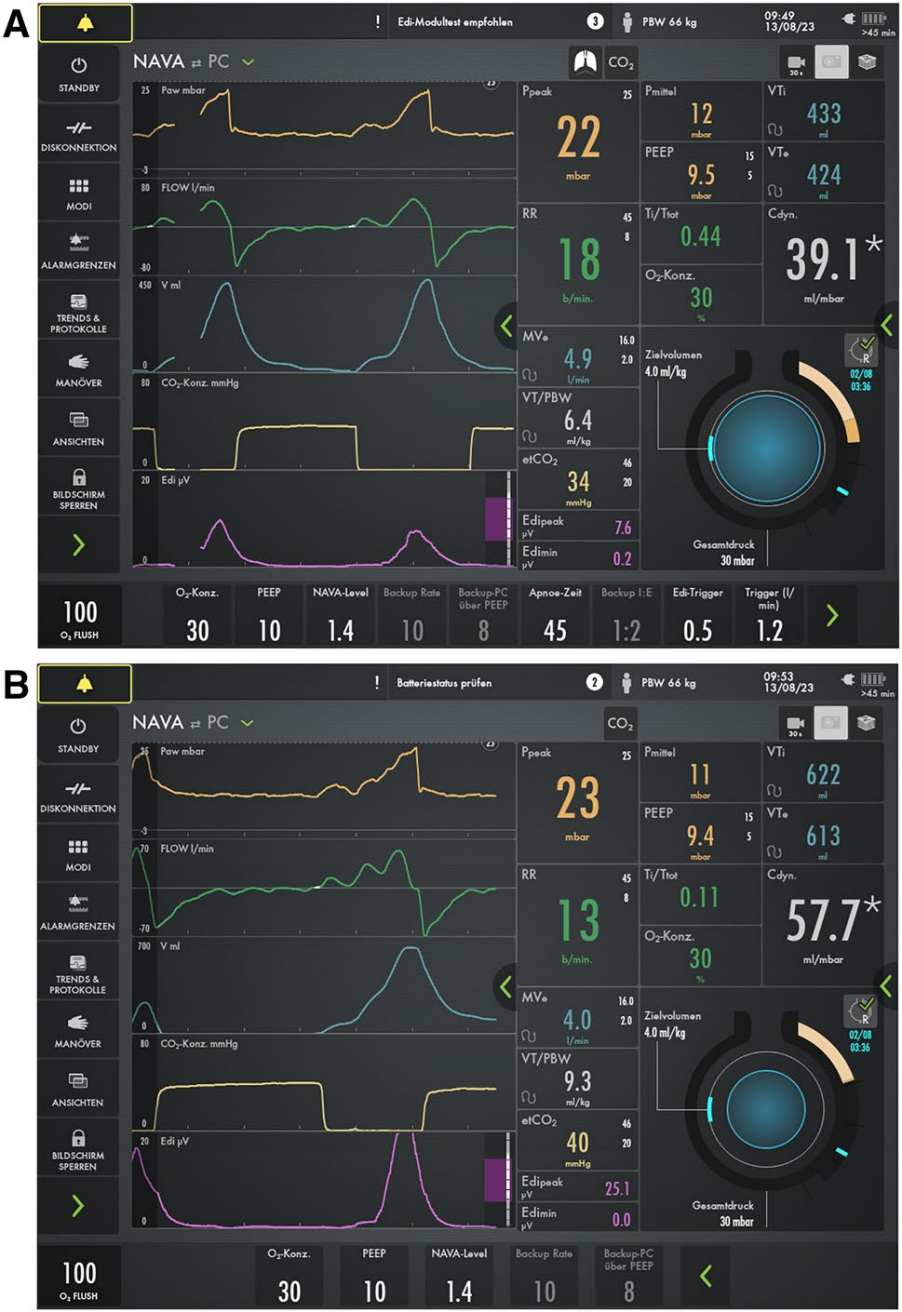
**a** before gas flow break **b** after gas flow break

The further course was complicated by an infected wound, remaining hematoma, and pneumatocele, all of which had to be surgically treated while still on ECMO. After 31 days of ECMO and ICU treatment, lung function had recovered so that spontaneous breathing could be established after reduction in sedation while still on VV-ECMO. When sedation was reduced, the patient gradually became conscious, however, due to severe critical illness acquired weakness syndrome, ventilation/carbon dioxide removal remained problematic for a prolonged period of time, while oxygenation was almost unimpaired [22]. Consequently, circuit blood flow could be reduced to 2.5–3.5 L/min, while the sweep-gas flow had to be remained between 2 and 4 L/min initially. Under these conditions, a protocolized gradual reduction in sweep-gas flow to 0 L/min was introduced to begin the final ECMO weaning step. However, due to an anxious patient, despite increased sedation, including the use of dexmedetomidine, adequate calming could not be achieved and the usual parameters to detect ECMO weaning failure (SpO2 < 88%, patient distress, pronounced tachypnea, pronounced tachycardia, and hypertension) were not conclusive. Due to our experience with NAVA during mechanical ventilation weaning, we attempted to use this mode during the final phase of ECMO weaning, that is, sweep-gas flow termination. The Edi peak was monitored and the ventilator settings were adapted throughout the weaning procedure. During NAVA-assisted weaning, we aimed to maintain the Edi peak generally within a range of 5–15 µV, with adjustments to the ventilator settings made if the Edi peak exceeded 15 µV. The Edi peak was used as a control parameter, and if it rose above 25 µV, despite ventilator adjustments, it was interpreted as diaphragm fatigue, leading to the immediate termination of the sweep-gas flow break as part of the individualized weaning protocol. Figure 2a shows an example of a ventilator graph during a sweep-gas flow of 6 L/min phase, with an Edi peak of 7.6 µV, and Fig. 2b shows the same patient during a phase of flow break phase with an Edi peak of 25.1 µV and the peak pressure terminated by the alarm setting immediately before the end of the sweep-gas flow break (Fig. 2 Ventilator graphics with NAVA a) before sweep-gas flow break b) after sweep-gas flow break). NAVA monitoring could effectively, conclusively, sooner than the usually used termination parameters, and independent of the patient’s fear, represent changes in diaphragm activation during sweep-gas flow break. This is well illustrated in Table 1 with the essential ventilation parameters of an exemplary weaning process of a gas flow of 0 L/min between 06:00 and 08:30 (Table 1 Sweep gas flow break from 06:00 to 08:30).With this protocol, the sweep-gas flow-free time could be continuously extended and ECMO was successfully removed on day 27 after admission without significant complications (Fig. 3).

**Table 1:** Sweep-gas flow break from 06:00 to 08:30

	MVi (lt/min)	RR spont	VT i max (ml)	Edi max µV	Heart rate (bpm)	Blood Pressure (mmHg)	SpO2 in %
06:00	9.8	20	428	8.7	104	129/63	100
06:30	10.44	22	421	9.6	99	123/60	100
07:00	10.3	20	335	13.6	105	130/60	100
07:30	11.63	18	629	11,6	107	138/56	100
08:00	10.86	18	765	17	105	132/63	100
08:30	10.59	26	755	20	108	115/54	100
09:00	10.4	20	305	15.8	106	133/61	100

The further weaning from mechanical ventilation went quickly and smoothly, all wounds healed by and by and were finally surgically covered. The patient was discharged 49 days after admission to a rehabilitation facility with mild swallowing difficulties and critical illness myopathy and polyneuropathy. The tracheostomy was covered with dressings and no abnormalities in gas exchange were observed without oxygen supply.

## Discussion

The case of a 27-year-old patient with a penetrating thoracic injury marked by evolving respiratory distress and the application of VV-ECMO as a rescue measure and weaning of the fully awake but very anxious patient presents a complex trajectory. Since there are probably as many different weaning procedures as ECMO centers around the world, but most centers do not disclose their weaning procedure, we cannot be sure. However, it is an irrefutable fact that there are no general principles on which we as a global ECMO treatment community have formally agreed. It starts with the fact that it is not clear whether weaning from mechanical ventilation or ECMO support comes first. What seems to be pretty consistently clear is that during lung recovery blood flow is reduced first (to what extent differs significantly) and the second and final step always becomes sweep-gas flow weaning. When the patient is able to maintain arterial carbon dioxide tension, pH, respectively, within the normal ranges for a significant amount of time (cut-off point for ‘significant’ again varies substantially) the ECMO support is removed.

This weaning approach, although successful in most cases, focusses almost entirely on the carbon dioxide removal capacity of the natural lung as the only success criterium [4, 25]. Only in very few procedural descriptions of mechanical factors such as work of breathing (WOB) or respiratory rate are mentioned, often as a sidenote or additional factors for successful termination of ECMO [19]. What is largely forgotten in this approach is that an increased WOB for increased respiratory minute volume, which must naturally occur when extracorporeal carbon dioxide removal is turned off, may not be feasible for a critically ill patient with ICUAWS (intensive care unit acquired weakness syndrome) and associated diaphragmatic weakness. Even evaluation of esophageal pressure (PES) as an effort signal may not always provide sufficient information to detect weaning failure due to ICUAWS [7, 13]. Problematic in this approach is that weaning failure need not be obvious at all, but may occur subclinical through increased respiratory muscle activation. However, this undetected increase in breathing force can cause problems such as renewed gas exchange failure (which may then be interpreted as recurrent pneumonia) or respiratory exhaustion leading to prolonged weaning. This subclinical ventilatory insufficiency can be approached using surrogate parameters such as WOB and P0.1 or, as a very nonspecific parameter, the respiratory rate, although these parameters are, except for the respiratory rate, rarely available continuously.

However, with NAVA, it is possible to visualize and assess the diaphragmatic force throughout the distance of each breath, as well as the neuromuscular coupling, even before changes in VT (tidal volume), respiratory rate, or esophageal pressure become clinically visible (illustrated in Table 1). Edi peak enables the therapist to decide whether gas exchange and ventilatory function are sufficient for decannulation or are at the expense of diaphragmatic force and problems are to be expected or whether gas exchange is accompanied by low (physiologic) activity of the diaphragm. In this way, the best initial situation for decannulation can be created not only for gas exchange but also for the diaphragm, avoiding respiratory distress and even diaphragmatic injury.

During the final step of the VV-ECMO weaning process, Edi peak can indirectly anticipate diaphragmatic fatigue (increase in Edi peak during the weaning process above a value of 25 µV) [23]. From our perspective, the initiation of VV-ECMO weaning is currently the optimal time to establish NAVA, as this stage provides the most stable and comparable conditions. By adjusting the NAVA level based on the Edi peak, within an assumed optimal range of 5–15 µV, future approaches could aim to gradually extend the gas flow pause, leading to a more refined weaning process and potentially reducing the overall weaning duration. While this method is still experimental and the optimal Edi peak range (5–15 µV) is derived from various studies—only one of which directly relates to the Edi peak in adult VV-ECMO weaning—in this case it serves as a practical reference for adjusting the ventilator settings [2, 10, 14, 16].

In this case report, we present the use of NAVA to monitor the VV-ECMO weaning process, which contributed to the successful removal of the ECMO support, for the first time. This case introduces a weaning approach that not only focusses on carbon dioxide removal as a marker of readiness for decannulation but introduces a continuous evaluation of diaphragm strength or fatigue in the weaning process and facilitated the targeted integration of ECMO weaning with ventilation settings. Since this is one of the few descriptions of this approach, naturally more evidence must be generated in larger cohorts.

## Conclusion

In conclusion, this case demonstrates the successful use of NAVA to facilitate the weaning process from VV-ECMO in a critically ill patient with a penetrating thoracic injury. The integration of NAVA allowed for continuous monitoring of diaphragm activity via the Edi peak, providing an individualized approach to adjusting ventilator support. By maintaining the Edi peak within a targeted range of 5–15 µV, we were able to optimize the weaning process and prevent diaphragm fatigue, as values exceeding 25 µV indicated the need to pause the weaning attempt. Although the use of NAVA in this context remains experimental, this approach highlights the potential for reducing weaning duration and improving patient outcomes. Further studies are needed to validate these findings and explore the correlation between diaphragm movement and Edi peak during the weaning process.

## Supplementary Information

Below is the link to the electronic supplementary material.

Supplementary file1 (DOCX 98 KB)

## Data Availability

All data generated or analyzed during this study are freely available on PubMed (https://pubmed.ncbi.nlm.nih.gov/). The specific datasets and references used in the current study can be accessed by searching the corresponding PMIDs provided within the manuscript.
