# Supplementary material for: ECMO-weaning facilitated by neurally adjusted ventilatory assist (NAVA): a case for principal clarification
Source: J Artif Organs. 2024 Dec 13;28(3):462–7. doi: 10.1007/s10047-024-01484-6 (PMC12373675; doi:10.1007/s10047-024-01484-6)
Supplement: Supplementary file 1 — Supplementary file1 (DOCX 98 KB) [file 10047_2024_1484_MOESM1_ESM.docx]

Sup. Tab. 2.

ECMO, ABG and Ventilator-Settings during NAVA-Trial.

Sup. Graphic 1.


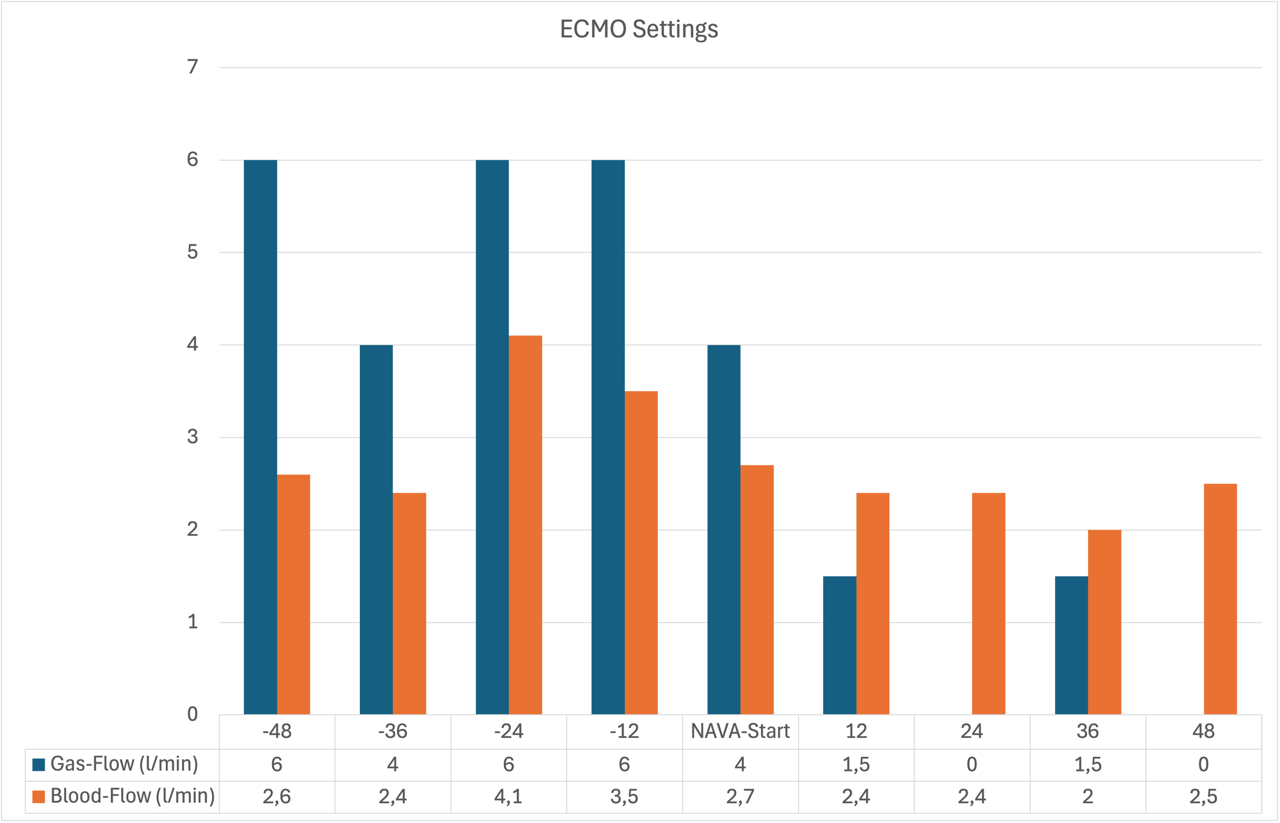


ECMO and Ventilator-Settings during NAVA-Trial.
